# The scale and timing of child protection system contacts from 0-18 years: a study of 1.9 million Australian children

**DOI:** 10.23889/ijpds.v9i5.1678

**Published:** 2024-09-18

**Authors:** Rhiannon Pilkington, Kathleen Falster, Mark Hanly, Alicia Montgomerie, Tasnia Ahmed, BJ Newton, Ben Edwards, Raghu Lingam, Anthony Shakeshaft, Michelle Cretikos, Jessica Stewart, Katherine Hawkins, Kitty McLean, John Lynch

**Affiliations:** 1 University of Adelaide; 2 UNSW; 3 ANU; 4 UQ; 5 NSW Ministry of Health; 6 NSW Department of Communities and Justice; 7 SA Department of Human Services; 8 SA Department for Child Protection

## Introduction

To prevent abuse, neglect and poor outcomes for children and families, including child protection intervention, maltreatment, and removals, it is critical to understand the scale and timing of child protection system contacts to identify opportunities for early prevention.

## Objectives and Approach

We quantified the cumulative incidence of child protection contacts during childhood, including initial reports, screened-in reports, investigations, substantiations and removal into out-of-home-care, among all children in two Australian states. We used child protection system data linked for the South Australian (SA) BEBOLD and NSW Child E-Cohort data platforms as the numerator, and census data for the denominator, for 571,497 SA children (birth-years 1991-2019) and 1,362,505 NSW children (birth-years 2005-2018).

## Results

Of children born in 2005 (NSW SA): Two in five children were reported to child protection by age 14; one in ten children were the subject of a child protection investigation in NSW and SA; one in twelve children in NSW and one in 17 in SA, were substantiated at least once; and one in 35 children in NSW and one in 40 in SA were removed into out-of-home care at least once by age 14. Approximately half of all types of child protection contact first occurred by the child’s fourth birthday.

## Conclusions/Implications

The scale of lifecourse child protection contact underscores the opportunities for early health and social supports to prevent child maltreatment and escalating child protection intervention. Child protection reports also represent an asset to generate public health intelligence to inform early prevention.

